# Bifunctional Electrocatalysts Based on Mo-Doped NiCoP Nanosheet Arrays for Overall Water Splitting

**DOI:** 10.1007/s40820-019-0289-6

**Published:** 2019-07-13

**Authors:** Jinghuang Lin, Yaotian Yan, Chun Li, Xiaoqing Si, Haohan Wang, Junlei Qi, Jian Cao, Zhengxiang Zhong, Weidong Fei, Jicai Feng

**Affiliations:** 1 State Key Laboratory of Advanced Welding and Joining, Harbin Institute of Technology, Harbin, 150001 People’s Republic of China; 2 School of Materials Science and Engineering, Harbin Institute of Technology, Harbin, 150001 People’s Republic of China; 3 MIIT Key Laboratory of Critical Materials Technology for New Energy Conversion and Storage, State Key Laboratory of Urban Water Resource and Environment, School of Chemistry and Chemical Engineering, Harbin Institute of Technology, Harbin, 150001 People’s Republic of China

**Keywords:** Water splitting, Bifunctional electrocatalyst, Electronic structure, Freestanding, Metal phosphides

## Abstract

Rational design of efficient bifunctional electrocatalysts is highly imperative but still a challenge for overall water splitting. Herein, we construct novel freestanding Mo-doped NiCoP nanosheet arrays by the hydrothermal and phosphation processes, serving as bifunctional electrocatalysts for overall water splitting. Notably, Mo doping could effectively modulate the electronic structure of NiCoP, leading to the increased electroactive site and improved intrinsic activity of each site. Furthermore, an electrochemical activation strategy is proposed to form Mo-doped (Ni,Co)OOH to fully boost the electrocatalytic activities for oxygen evolution reaction. Benefiting from the unique freestanding structure and Mo doping, Mo-doped NiCoP and (Ni,Co)OOH show the remarkable electrochemical performances, which are competitive among current researches. In addition, an overall water splitting device assembled by both electrodes only requires a cell voltage of 1.61 V to reach a current density of 10 mA cm^−2^. Therefore, this work opens up new avenues for designing nonprecious bifunctional electrocatalysts by Mo doping and in situ electrochemical activation.

**Electronic supplementary material:**

The online version of this article (10.1007/s40820-019-0289-6) contains supplementary material, which is available to authorized users.

## Highlights

- Freestanding Mo-doped NiCoP nanosheets are designed as bifunctional electrocatalysts for overall water splitting.
- Remarkable electrocatalytic performances are achieved by Mo doping, where a low-water-splitting voltage of 1.61 V at 10 mA cm^−2^ is obtained.

**Electronic supplementary material:**

The online version of this article (10.1007/s40820-019-0289-6) contains supplementary material, which is available to authorized users.

## Graphical abstract

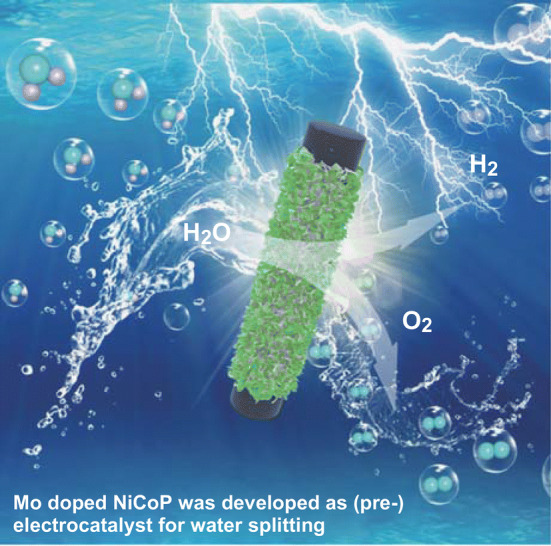

## Introduction

Electrochemical water splitting, consisting of oxygen evolution reaction (OER) and hydrogen evolution reaction (HER), is a promising approach to generate sustainable H_2_ from water [1–4]. Even though Ir-/Ru-based oxides and Pt catalysts show excellent electrocatalytic performances for OER and HER, the high cost and the scarcity of these noble catalysts greatly hinder their practical applications [5–8]. When different electrodes are used for a single water-splitting device, the incompatibility and different reaction kinetics in OER and HER may lead to inferior efficiency [9–11]. Thus, it is highly imperative to design low-cost and high-efficiency electrocatalysts for HER and OER simultaneously.

Among various alternative materials, transition metal phosphides (TMPs) have gained considerable attention, owing to the suitable *d*-electron configuration and rich chemical states [12–14]. In particular, NiCoP with the enhanced electrical conductivity and the synergistic effect from Ni and Co shows good catalytic performances for overall water splitting [15, 16]. To further improve the catalytic activity, considerable efforts have been devoted to increasing the number of active sites and improving the intrinsic activity of each site [17]. On the one hand, nano-/microstructure design has been conducted to increase the number of active sites [18–20]. However, most of current works focus on preparing powder-formed electrocatalysts, and the possible aggregation and involved polymeric binders may block the active sites and hinder the electron transfer [19, 20]. To solve this concern, a promising strategy is to develop freestanding arrays directly on conductive substrates, thereby assembling a binder-free electrode, which favors gas evolution and boosts electron transfer [21, 22]. However, simply increasing surface areas could not primarily change the intrinsic activity of each site. On the other hand, the element doping is a promising strategy to essentially improve the intrinsic activity of active sites, which could modify the electronic structure and accelerate the reaction kinetics [23, 24]. For example, O, Fe, and carbon dot-doped NiCoP have been fabricated, which showed enhanced electrocatalytic performances [25–27]. According to the reported research [28], Mo-based phosphides show the best HER performances among various binary phosphides. In this case, it is expected that the doping Mo on freestanding NiCoP nanosheets could achieve the improvement in electrocatalytic performances.

**Scheme 1:**
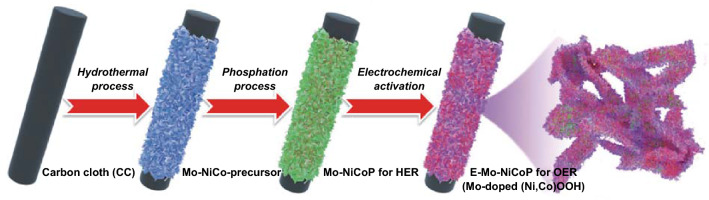
Schematic fabrication process of Mo-doped NiCoP and E-Mo–NiCoP nanosheet arrays directly on carbon cloth

Motivated by the above thoughts, novel Mo-doped NiCoP nanosheet arrays are rationally designed as bifunctional electrocatalysts for overall water splitting. As shown in Scheme 1, Mo-doped NiCoP nanosheets are fabricated by the hydrothermal and phosphation processes. Further, considering that TMPs are not stable and the corresponding oxides/hydroxides are the actual electroactive species in OER [29, 30], we also propose an electrochemical activation strategy to in situ formed Mo-doped (Ni,Co)OOH arrays from Mo-doped NiCoP. As bifunctional electrocatalysts, our designed Mo-doped NiCoP exhibits the following advantages: (1) the freestanding arrays directly on carbon cloth could maximize active surface areas and shorten ion diffusion length. (2) Mo doping could effectively modulate the electronic structure, leading to the increased electroactive site and accelerated the Volmer step and OH^−^ adsorption. (3) In situ formed Mo-doped (Ni,Co)OOH could fully boost the electrocatalytic activities for OER. Consequently, Mo-doped NiCoP arrays show the competitive HER performances, featured by the overpotential of 76 mV at 10 mA cm^−2^. Furthermore, Mo-doped (Ni,Co)OOH arrays show a significant increase in the OER activity. In addition, an overall water-splitting device assembled by both electrodes only requires 1.61 V cell voltage to reach 10 mA cm^−2^.

## Experimental Section

### Synthesis of Mo-Doped NiCoP Nanosheets on Carbon Cloth

Prior to synthesis, carbon cloth (2 × 5 cm^2^) was treated by concentrated nitric acid at 100 °C for 3 h, then washed with deionized water and ethanol and dried in the air [31]. The whole synthesis process is shown in Scheme 1. At first, Mo-doped NiCo precursors were synthesized by the hydrothermal process. Typically, NiCl_2_·6H_2_O (4 mmol), CoCl_2_·6H_2_O (4 mmol), and Na_2_MoO_4_·6H_2_O were dissolved in 60 mL deionized water to form a pink solution. To optimize the Mo doping, different contents of Na_2_MoO_4_·6H_2_O (0, 0.1, 0.2, 0.3, and 0.4 mmol) were conducted to synthesize various Mo–NiCo precursors (shortly named as Mo–NiCo-precursor-1–4). The pink solution with treated carbon cloth was transferred to 100-mL Teflon-lined stainless-steel autoclave, heated at 100 °C for 6 h. After that, the precursors were collected, washed with deionized water and ethanol for several times, and dried in vacuum overnight.

Secondly, the Mo–NiCo precursors were converted to corresponding Mo–NiCoP by the phosphation process (shortly named as Mo–NiCoP-1–4). The as-prepared precursors and 0.5 g NaH_2_PO_2_·H_2_O were placed at both ends of a tubular furnace, and NaH_2_PO_2_·H_2_O was at the upstream side. The phosphation process was conducted at 300 °C for 1 h with heating rate of 5 °C min^−1^ under continuous Ar flow.

Finally, the core-branched Mo–(Ni,Co)OOH arrays were in situ transformed from Mo–NiCoP by the electrochemical activation in the three-electrode cell (shortly named as E-Mo–NiCoP-1–4). The electrochemical activation was conducted by cyclic voltammetry (CV) under a potential window of 0–1 V versus Hg/HgO in 6 M KOH at the scan rate of 10 mV s^−1^. Different CV cycles (0, 12, 25, 50, and 75) were applied to optimize the OER performances of obtained samples.

### Material Characterizations

The structure and morphology of as-obtained samples were analyzed by scanning electron microscopy (SEM, Helios NanoLab 600i) and transmission electron microscopy (TEM, Tecnai G2 F30). The phases were characterized by X-ray diffraction (XRD, D8 Advance). The chemical states were verified by X-ray photoelectron spectroscopy (XPS, Thermo Fisher) measurements.

### Electrochemical Measurements

All tests were performed on the CHI760E electrochemical station. Both HER and OER were measured in a three-electrode configuration using 1 M KOH as electrolyte. The carbon cloth with active materials was directly employed as the working electrode in electrochemical measurement, while the graphite bar and Hg/HgO electrode were used as the counter and reference electrodes, respectively. The polarization curves were recorded at 2 mV s^−1^ and were compensated with iR correction. All potentials were converted into the reversible hydrogen electrode (RHE). Electrochemical impedance spectroscopy (EIS) tests were conducted in the frequency range from 0.1 Hz to 100 kHz. The mass loading of NiCoP or Mo-doped NiCoP on carbon cloth was about 2 mg cm^−2^. The reference electrodes of Pt/C and RuO_2_ with 2 mg cm^−2^ mass loading were also prepared on carbon cloth, and the prepared method was according to previously reported researches [32, 33].

## Results and Discussion

### Structural and Morphological Characterizations

**Fig. 1:**
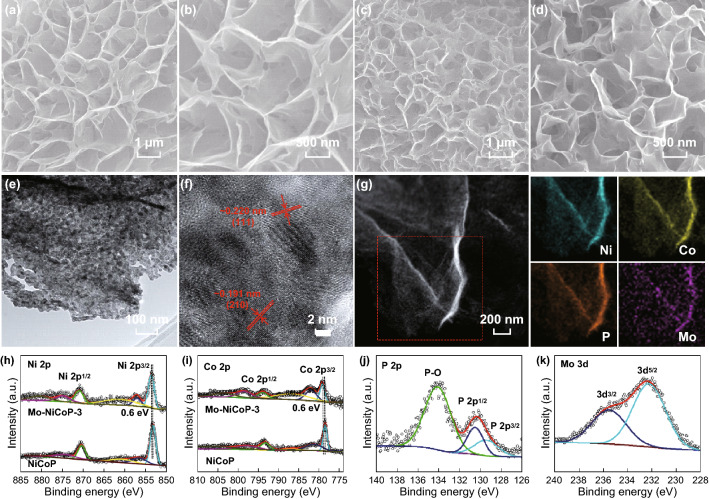
SEM images of **a**, **b** NiCoP and **c**, **d** Mo–NiCoP-3. **e** TEM, **f** HRTEM, and **g** the corresponding element mapping in Mo–NiCoP-3. XPS **h** Ni 2*p* and **i** Co 2*p* spectra in NiCoP and Mo–NiCoP-3. XPS **j** P 2*p* and **k** Mo 3*d* spectra in Mo–NiCoP-3

The Mo-doped NiCoP nanosheets are synthesized by the transformation from Mo–NiCo-precursor using a vapor phosphation process. SEM images in Fig. S1 show that after introducing Mo cation, all obtained samples maintain the nanosheet structures with smooth surfaces directly on carbon cloths. After phosphation process, the original smooth nanosheets become rugged and porous for all samples (Fig. S2 and Fig. 1a–d). XRD patterns of all Mo–NiCoP samples in Fig. S3 demonstrate that all the phases could be indexed to NiCoP (JCPDS card No. 71-2336), except for the peak from carbon cloth. It demonstrates that Mo doping does not change the crystal structure of NiCoP. Further, the porous nanostructure in Mo–NiCoP-3 could be also confirmed by TEM image in Fig. 1e. Undoubtedly, the porous structure is beneficial for charge transfer efficiency and mass transport during electrocatalytic process. The corresponding high-resolution TEM image in Fig. 1f visualizes the well-defined lattice fringes with interplanar distances of about 0.191 and 0.220 nm, which corresponds well to the (210) and (111) planes of NiCoP. Further, the corresponding element mapping in Fig. 1g demonstrates that the Ni, Co, Mo, and P elements are uniformly distributed in the nanosheets. For comparison, pure NiCoP nanosheets are also characterized by TEM in Fig. S4. It can be found that pure NiCoP possesses the similar nanostructure and crystal structure with Mo–NiCoP-3.

To investigate the chemical states and electronic structures, XPS measurements are taken to comparably analyze the obtained samples. As for all Mo–NiCoP samples in Fig. S5, the binding energies of Ni 2*p* have been positive-shifted with gradually increasing Mo doping. As for Ni 2*p* in Mo–NiCoP-3 (Fig. 1h), the peaks at 853.7 and 870.9 eV are from the Ni^*δ*+^ in NiCoP, while the peaks at 857.0 and 874.9 eV are attributed to oxidized Ni species, as well as two peaks from the satellites [34, 35]. In Co 2*p* spectra (Fig. 1i), the Co 2*p*_1/2_ region shows the two peaks at 793.8 and 798.4 eV as well as a satellite peaks. For Co 2*p*_3/2_, the peak at 779.0 eV is attributed to Co–P bond, while the peak at 781.8 is assigned to Co-oxidized state [35, 36]. As for P 2*p* in Fig. 1j, the peaks at 130.5, 129.4, and 134.1 eV are from P 2*p*_1/2_, P 2*p*_3/2_, and surface-oxidized P–O species [37]. In addition, the Mo 3*d*_3/2_ at 235.5 eV and Mo 3*d*_5/2_ at 232.2 eV in Mo 3*d* spectrum (Fig. 1k) demonstrate the main presence of Mo^6+^ [24]. Thus, Mo element exists in the form of atomic substitution by Ni or Co. To investigate the Mo mass loading in each samples, we also conducted the inductively coupled plasma optical emission spectroscopy (ICP-OES) analysis. The Mo mass loadings for Mo–NiCoP-1–4 are 0.078, 0.155, 0.199, and 0.257 mg cm^−2^ by ICP analysis. According to previous researches [25, 38, 39], the XPS peak shift shows the changes of electronic configuration and electron transfer between different cations, further implying the electronic interactions after Mo doping. By comparing Ni 2*p* and Co 2*p* spectra in NiCoP and Mo–NiCoP-3, it can be found that the binding energies have been positive-shifted by about 0.6 eV. It demonstrates that Mo doping could effectively modify the electronic structure of Ni and Co centers in obtained samples [24, 39]. Such positive shift in binding energies could be attributed to electron transfer owing to the presence of Mo^6+^, suggesting the electron transfer from Mo atoms to nearby atoms [40, 41]. The fast electron transfer brought by Mo doping could be beneficial for OER and HER process [40, 42].

### The Effect of Mo Doping on HER Performances

**Fig. 2:**
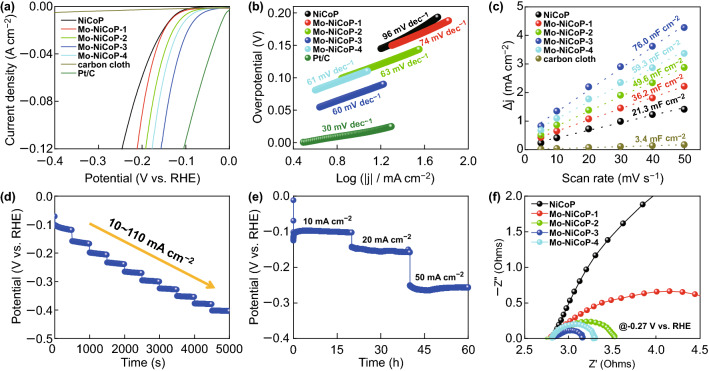
**a** Polarization curves, **b** the corresponding Tafel slope plots, and **c** the current density against scan rates of obtained samples. **d** Consecutive multi-step chronoamperometric tests for Mo–NiCoP-3 (without iR correction). **e** Chronopotentiometry measurements for Mo–NiCoP-3 (without iR correction). **f** The Nyquist plots of obtained samples

The electrocatalytic activity of obtained samples toward the HER is firstly measured by linear scan voltammetry (LSV) in 1 M KOH. As shown in Fig. 2a, the Mo-doped NiCoP samples show the better HER performances than that of NiCoP electrode. In particular, Mo–NiCoP-3 electrode shows the low overpotentials of 76, 121, and 148 mV at the current densities of 10, 50, and 100 mA cm^−2^, comparable to that of Pt/C, demonstrating that Mo doping could effectively enhance the HER activity. To avoid the influence of surface area, the LSV curves of HER are normalized by the electrochemical surface area (ECSA) in Fig. S8. For HER, Mo–NiCoP-3 sample shows the better performances than that of NiCoP, which demonstrates that Mo doping could effectively increase intrinsic activities. As further compared with recently reported electrocatalysts in Table S1, our synthesized Mo–NiCoP shows the competitive HER activities. To investigate the HER kinetics, the Tafel slope is calculated in Fig. 2b. It can be found that Mo–NiCoP-3 electrode shows the lowest Tafel slope of 60 mV dec^−1^, which is smaller than that of pure NiCoP (96 mV dec^−1^), and is close to that of Pt/C (30 mV dec^−1^). While the Tafel slope is in the range of 40–120 mV dec^−1^, HER reaction in Mo–NiCoP-3 follows the Volmer–Heyrovsky mechanism, and the Volmer step is the rate-determining step [40]. The lower Tafel slope of Mo–NiCoP-3 suggests the decrease in charge transfer resistance during HER process and faster HER kinetics, which is favorable to achieve higher gas evolution rates in practical applications [37, 39]. According to previous researches [1, 6], the water dissociation process for HER in alkaline media may require an additional energy barrier, which would govern the overall reaction rate. And the balanced H*/OH* adsorption abilities are also required for HER in alkaline media [1, 3]. Consequently, it is vital to optimally balance water dissociation and H*/OH* adsorption abilities for electrocatalytic materials. According to previous research [12], Ni site has good water dissociation ability, while Mo site shows superior adsorption capability toward H. Further, appropriate Mo doping content in Mo–NiCoP may tune the Gibbs free energies of hydrogen adsorption, which may be another reason for the enhanced HER performances [42]. Therefore, Mo-doped NiCoP shows a low overpotential and fast HER kinetics in 1 M KOH.

To bridge the structural properties and intrinsic activity, the electrochemical surface area (ECSA) was estimated by the electrochemical double-layer capacitance (*C*_dl_). Based on the cyclic voltammograms (CVs) versus the scan rate in Fig. S9, the *C*_dl_ values are calculated in Fig. 2c. Obviously, Mo–NiCoP-3 electrode shows the high *C*_dl_ value up to 76.0 mF cm^−2^, which is much higher than that of pure NiCoP (21.3 mF cm^−2^). This comparison suggests that Mo doping in NiCoP nanosheet arrays could greatly enhance the active sites, leading to performances in HER. Figure 2d shows the consecutive multi-step chronoamperometric tests for Mo–NiCoP-3, where the potential remains steady in each step. It suggests the excellent mass transport and mechanical properties of Mo–NiCoP-3 electrode in HER tests. The durability of Mo–NiCoP-3 electrode in HER was tested in 10, 20, and 50 mA cm^−2^ for each 15 h, as shown in Fig. 2e. Obviously, in each current density, the potentials of Mo–NiCoP-3 electrode remain stable with small degradation. In addition, SEM, XRD, and TEM results in Fig. S10 show that the morphology, structure, and phases of Mo–NiCoP-3 are well retained after longtime HER tests, again demonstrating the good durability. To investigate the charge transfer kinetics during HER, the EIS tests were conducted at − 0.27 V versus RHE. The Nyquist plots in Fig. 2f unravel that Mo–NiCoP-3 electrode shows the lowest charge-transfer resistance (*R*_ct_), suggesting the fastest electron transport for HER [39, 43]. It may be contributed to the modified electronic structure of NiCoP by Mo doping. And, the lowest *R*_ct_ further confirms the fastest evolution kinetics and smallest Tafel slop in HER.

### The Effect of Electrochemical Activation on OER Performances

**Fig. 3:**
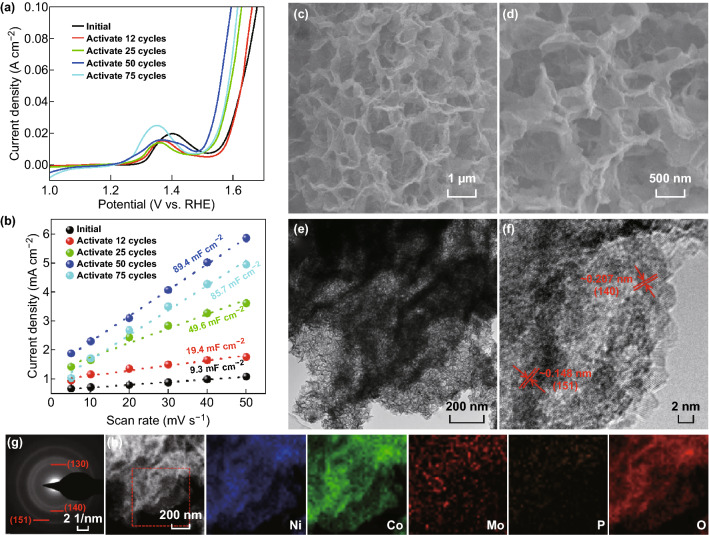
**a** Polarization curves and **b** the current density against scan rates of E-Mo–NiCoP-3 with different activation cycles as indicated. **c**, **d** SEM, **e** TEM, **f** HRTEM, **g** SAED, and **h** the corresponding element mapping of E-Mo–NiCoP-3

As described in Introduction, metal phosphides are not stable during electrochemical tests. Therefore, in situ electrochemical activation by CV tests was applied to investigate the changes of the nanostructures, phases, and electrochemical performances. Figure 3a shows the polarization curves of E-Mo–NiCoP-3 with different activation cycles. And the corresponding *C*_dl_ values are also calculated in Fig. 3b. With the increase in activation cycles, it can be found that the overpotential is gradually reduced and the *C*_dl_ is gradually increased. Further, the *R*_ct_ values in Fig. S12a show the similar trend. It suggests that the electrochemical activation could boost the catalytic activities and enhance the electroactive sites for OER. After the CV cycles reach 75, the *C*_dl_ value is slightly reduced. Thus, the optimum CV cycles are determined to be 50 cycles. Further, E-Mo–NiCoP-3 sample was further characterized by SEM and TEM in Fig. 3c–f. Obviously, the nanosheet structures and morphologies are well maintained after electrochemical activation. However, the nanosheets become frizzier and rougher. From the TEM images in Fig. 3e, it could be found that a large number of ultra-nanosheets are formed, which may be due to the electrochemically induced ion-exchange process [44]. As illustrated in Scheme 1, the core-branched nanosheet arrays are formed after electrochemical activation. The phases of all E-Mo–NiCoP samples are investigated by XRD in Fig. S15a. After electrochemical activation, all the peaks in all E-Mo–NiCoP samples (except for the peak from carbon cloth) could be indexed to (Ni,Co)OOH phase from the partial substitution Co ions by Ni ions in the CoOOH (JCPDS Card No. 26-0480). And no metal phosphides could be found in XRD patterns at all. The HRTEM image in Fig. 3f shows the lattice fringe spacings of 0.148 and 0.207 nm, corresponding to (151) and (140) planes of (Ni,Co)OOH phase. Further, the selected area electron diffraction pattern in Fig. 3g shows several diffraction rings, which could be indexed to (130), (140), and (151) planes of (Ni,Co)OOH, coinciding with the XRD results. Further, the element mapping images in Fig. 3h show that the P element has been greatly reduced, which may be leaked during electrochemical activation. And it can be found that Ni, Co, Mo, and O elements are uniformly distributed over the nanosheets of E-Mo–NiCoP-3.

As for all E-Mo–NiCoP samples (Fig. S14a), it can be found that the binding energies for Ni 2*p* have been positive-shifted with gradually increasing Mo doping. It demonstrates that Mo doping could effectively modify the electronic structure of Ni centers in obtained samples. And no obvious P 2*p* signal could be found in all E-Mo–NiCoP samples **(**Fig. S14b), which suggests that P element has been leaked during electrochemical activation. Detailed XPS analysis on E-NiCoP-3 is shown in Fig. S15b-f. The energy separation up to about 17.6 eV in Ni 2*p* spectrum demonstrates the formation of NiOOH (Fig. S15b) [45]. As for Co 2*p* in Fig. S15c, there are obvious satellite signals, suggesting the presence of low-spin Co^3+^ state [45]. As shown in Fig. S15d, no obvious P 2*p* signal could be found, even increasing the etching time. In addition, the Mo 3*d*_3/2_ and Mo 3*d*_5/2_ in Fig. S16e demonstrate the main presence of Mo^6+^. Further, three peaks (Fig. S15f) at 529.7, 531.5, and 532.6 eV are attributed to the lattice oxygen, hydroxyls, and oxygen defects [45, 46]. Thus, based on the above analysis, core-branched Mo-doped (Ni,Co)OOH arrays are in situ formed after electrochemical activation.

**Fig. 4:**
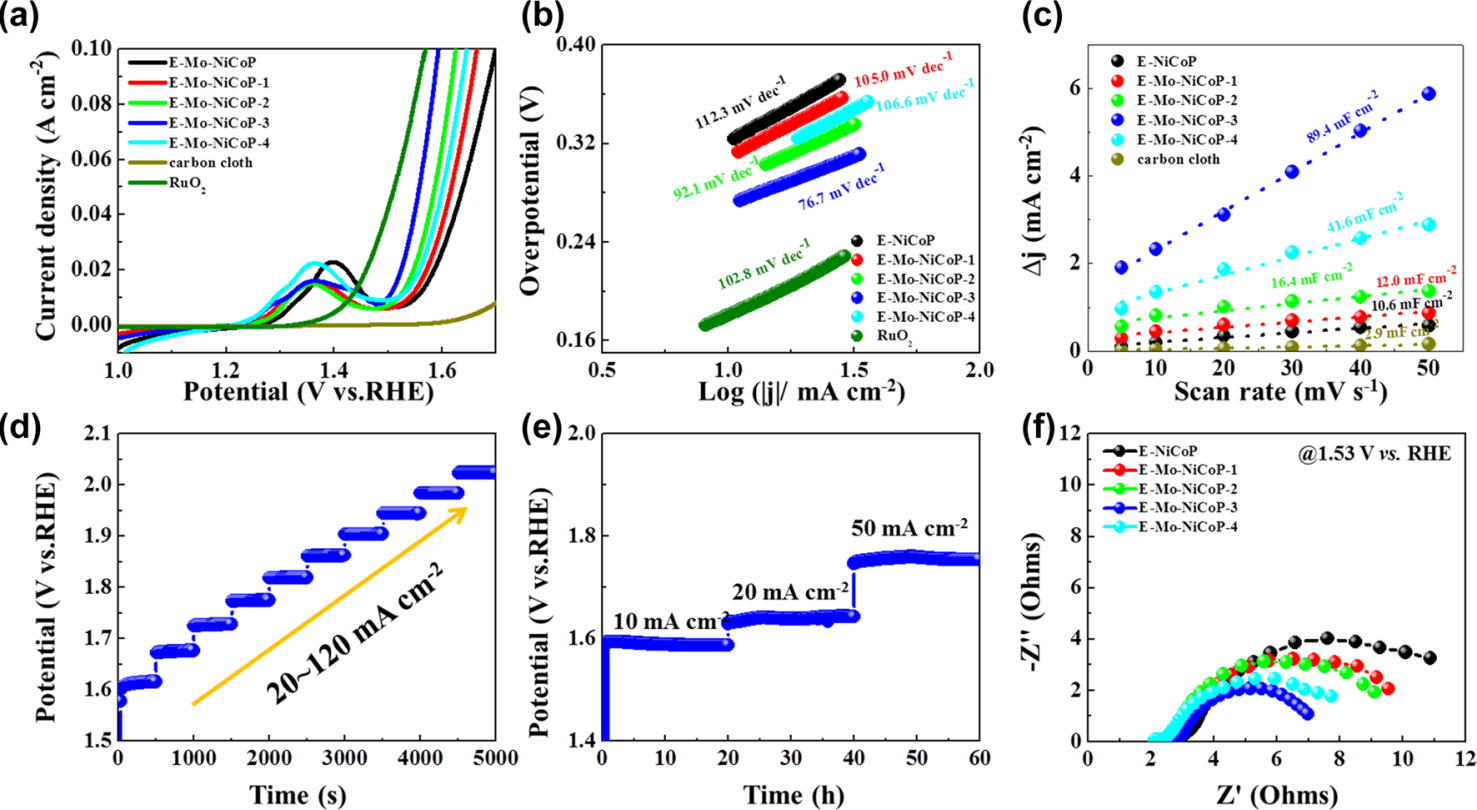
**a** Polarization curves, **b** the corresponding Tafel slope plots, and **c** the current density against scan rates of obtained samples after electrochemical activation. **d** Consecutive multi-step chronoamperometric tests for E-Mo–NiCoP-3 (without iR correction). **e** Chronopotentiometry measurements for E-Mo–NiCoP-3 (without iR correction). **f** The Nyquist plots of obtained samples after electrochemical activation

Therefore, all samples were treated by similar electrochemical activation (50 cycles) for fully boosting the OER performances. As shown in Fig. 4a, E-Mo–NiCoP-3 possesses the lower overpotentials than that of pure E-NiCoP, demonstrating that doping Mo in (Ni,Co)OOH arrays could also improve the OER activity. In particular, E-Mo–NiCoP-3 electrode shows the low overpotentials of 269, 328, and 364 mV at the current densities of 10, 50, and 100 mA cm^−2^, comparable to that of RuO_2_. Compared with pristine sample (E-NiCoP), Mo doping could reduce the overpotential by 60 mV. In the meanwhile, our synthesized E-Mo–NiCoP shows the competitive OER activities with reported nonprecious electrocatalysts (Table S2). To investigate the OER kinetics, the Tafel slope is calculated in Fig. 4b. The Tafel slope of E-Mo–NiCoP-3 is 76.7 mV dec^−1^, smaller than that of pure E-NiCoP (112.3 mV dec^−1^), demonstrating the faster OER kinetics after doping Mo. In general, the mechanism of OER in KOH electrolyte consists of a four-electron transfer process [30, 40]. The Tafel slope of E-Mo–NiCoP-3 is closed to a featured Tafel slope of 60 mV dec^−1^, suggesting a changed rate-determining step [38]. According to previous researches [24, 38, 39], cation incorporation could tune the reaction free energy of the rate-determining step and also lead to a more favorable electronic structure and provide more electroactive sites for OH^−^ adsorption. Thus, it might be another reason for the enhanced OER properties after Mo doping. The *C*_dl_ values in Fig. 4c toward OER also indicated the rich electroactive sites in E-Mo–NiCoP-3. The multi-step chronoamperometric tests in Fig. 4d proved to be the excellent mass transport of E-Mo–NiCoP-3 electrode in OER tests. The long-term stability toward OER in Fig. 4f further confirms the good durability of E-Mo–NiCoP-3. And SEM, TEM, and SAED images in Fig. S17 show that the morphology, structure, and phases of E-Mo–NiCoP-3 are well retained after longtime OER tests, again demonstrating the good stability. Again, similar to HER process, the Nyquist plots in Fig. 4f also show the lowest *R*_ct_ value in E-Mo–NiCoP-3, in accordance with highest kinetics induced by Mo doping.

**Fig. 5:**
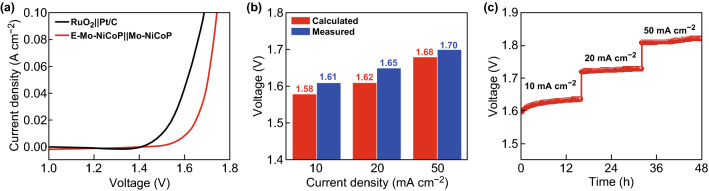
**a** Polarization curves of obtained samples for overall water splitting. **b** The comparison of measured and calculated voltages for overall water splitting. **c** Long-term stability at constant current densities of 10, 20, and 50 mA cm^−2^

Based on the above results, the freestanding E-Mo–NiCoP and Mo–NiCoP nanosheet arrays could serve as both the cathode and anode for overall water splitting in 1 M KOH. As shown in Fig. 5a, to achieve the current density of 10 mA cm^−2^, only 1.61 V is required. As shown in Fig. S18, the performance of E-Mo–NiCoP||Mo–NiCoP is much better than that without Mo doping. In particular, the performance of E-Mo–NiCoP||Mo–NiCoP is competitive with many reported electrocatalysts in Table S3. As shown in Fig. 5b, the measured voltages and calculated voltages are very close in different current densities, and the slight differences may be contributed to measurements in various testing systems. To calculate the faradaic efficiency, the quantity of gas is measured in Fig. S19. By comparing the measured and calculated gas amounts, the nearly 100% faradaic efficiency is found for E-Mo–NiCoP||Mo–NiCoP. Further, Fig. 5c shows that E-Mo–NiCoP||Mo–NiCoP cell shows the good stability at the current densities of 10, 20, and 50 mA cm^−2^.

## Conclusion

In summary, we have demonstrated cation doping and in situ electrochemical activation to boost the HER and OER performances of transition metal phosphides. Exemplified by freestanding NiCoP nanosheets on carbon cloth, Mo incorporation could effectively modulate the electronic configuration and increase the electroactive sites. Further, by electrochemical activation, the core-branched Mo-doped (Ni,Co)OOH arrays are formed, which could fully boost the OER activities. Consequently, the optimal Mo-doped NiCoP nanosheet arrays show the enhanced electrocatalytic performances as (pre-) electrocatalyst for efficient water splitting. Our work provides new insights for designing nonprecious bifunctional electrocatalysts by cation doping and in situ electrochemical activation.

## Electronic supplementary material

Below is the link to the electronic supplementary material.

Supplementary material 1 (PDF 1548 kb)
