# Supplementary material for: Bifunctional Electrocatalysts Based on Mo-Doped NiCoP Nanosheet Arrays for Overall Water Splitting
Source: Nanomicro Lett. 2019 Jul 13;11:55. doi: 10.1007/s40820-019-0289-6 (PMC7770736; doi:10.1007/s40820-019-0289-6)
Supplement: Supplementary file 1 — Supplementary material 1 (PDF 1548 kb) [file 40820_2019_289_MOESM1_ESM.pdf]

Supporting Information for

Bifunctional Electrocatalysts Based on Mo-doped NiCoP Nanosheet Arrays for Overall Water Splitting

Jinghuang Lin[1], Yaotian Yan[1], Chun Li[1], Xiaoqing Si[1], Haohan Wang[1], Junlei Qi[1,]*, Jian Cao[2], Zhengxiang Zhong[3], Weidong Fei[1], Jicai Feng[1]

[1]State Key Laboratory of Advanced Welding and Joining, Harbin Institute of Technology, Harbin 150001, People's Republic of China

[2]School of Materials Science and Engineering, Harbin Institute of Technology, Harbin 150001, People's Republic of China

[3]MIIT Key Laboratory of Critical Materials Technology for New Energy Conversion and Storage, State Key Laboratory of Urban Water Resource and Environment, School of Chemistry and Chemical Engineering, Harbin Institute of Technology, Harbin 150001, People's Republic of China

J. Lin and Y. Yan contributed equally to this paper

*Corresponding author. E-mail: jlqi@hit.edu.cn (Junlei Qi)

Supplementary Figures and Tables

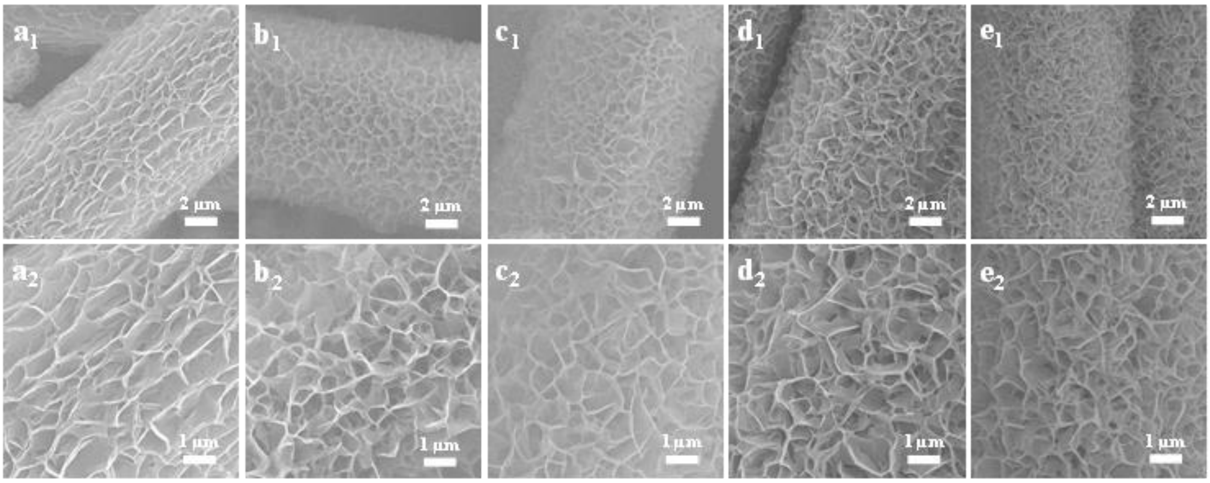

Fig. S1 SEM images of **a** NiCo-precursor, **b** Mo-NiCo-precursor-1, **c** Mo-NiCo-precursor-2, **d** Mo-NiCo-precursor-3, and **e** NiCo-precursor-4

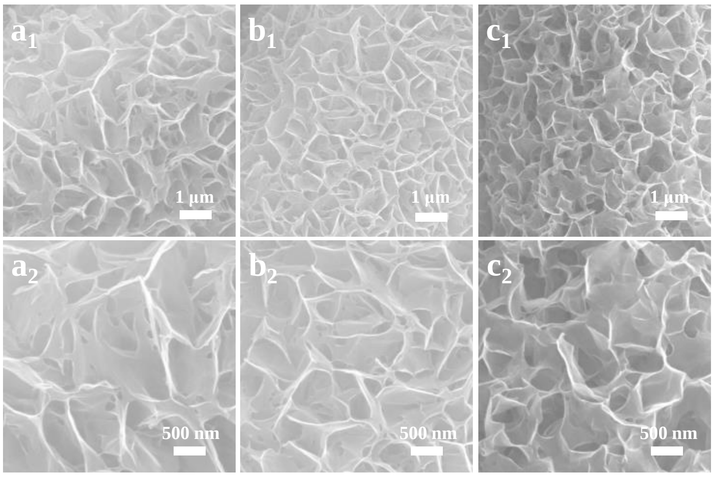

Fig. S2 SEM images of **a** Mo-NiCoP-1, **b** Mo-NiCoP-2, and **c** Mo-NiCoP-4

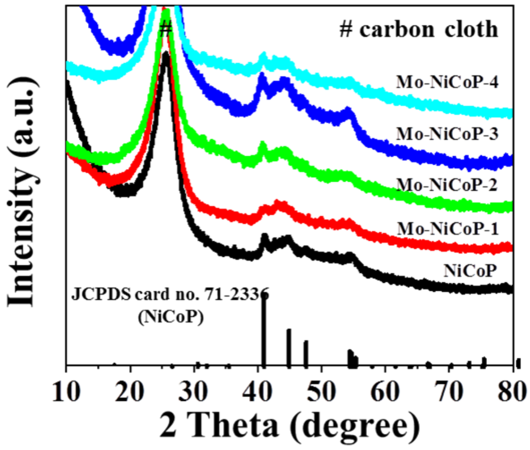

Fig. S3 XRD patterns of all Mo-NiCoP samples

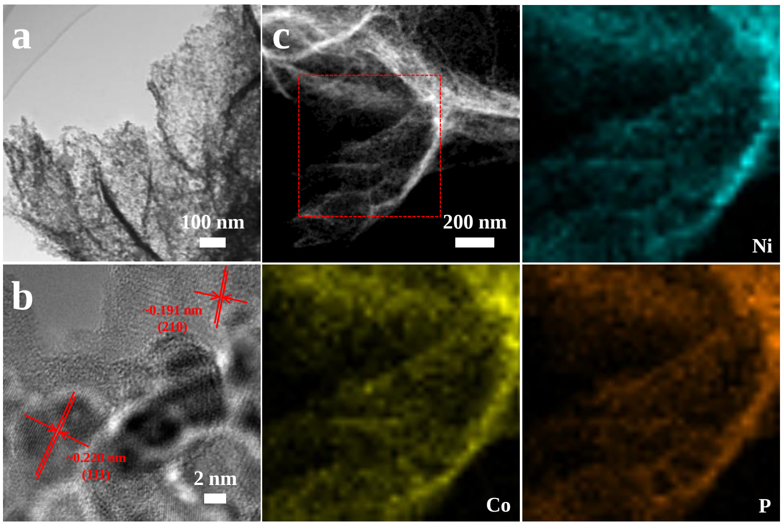

Fig. S4 a TEM, **b** HRTEM images of NiCoP, and **c** the correponding element mappings of NiCoP

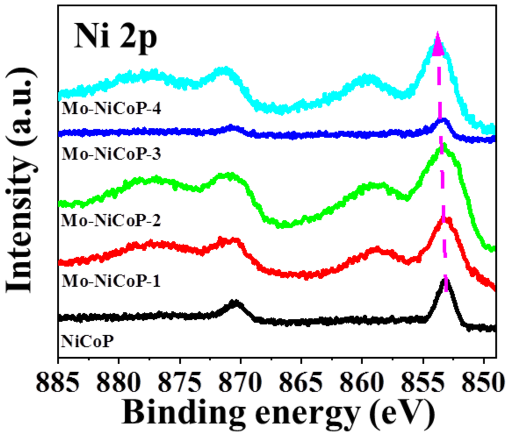

Fig. S5 XPS spectra of Ni 2p for all Mo-NiCoP

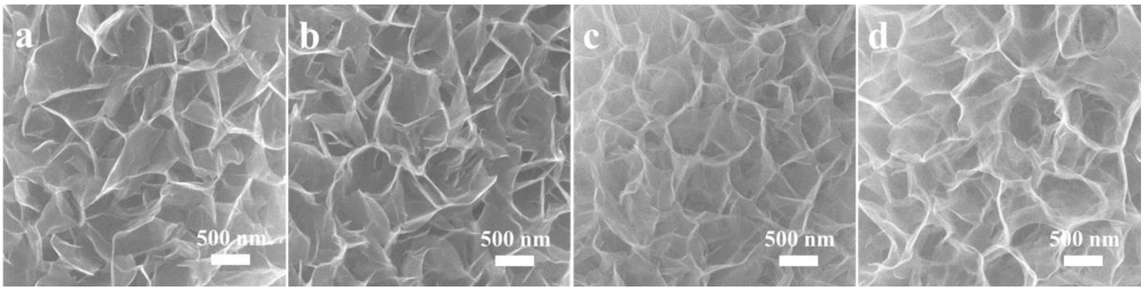

Fig. S6 SEM images of **a** NiCoP-1:7, **b** NiCoP-2:6, **c** NiCoP-6:2, and **d** NiCoP-7:1

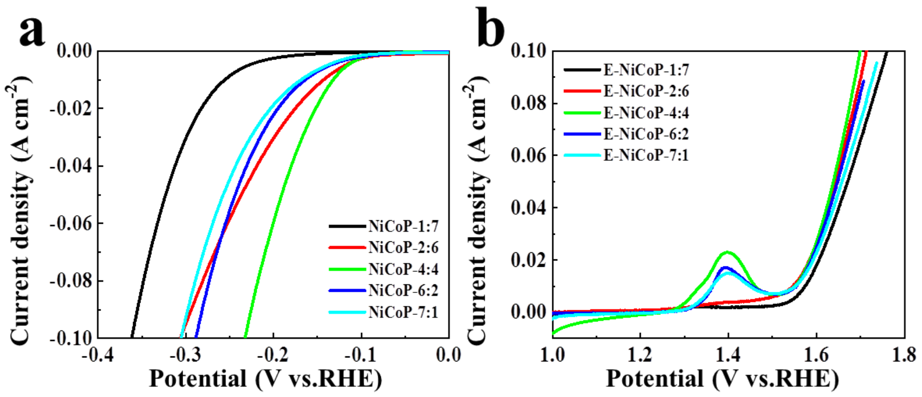

Fig. S7 The polarization curves of obtained samples for **a** HER and **b** OER

We conducted some experiments to tune the molar ratio of Co/Ni to enhance the activity of HER and OER. Different molar ratios of $NiCl_2·6H_2O$ and $CoCl_2·6H_2O$ (Ni:Co=1:7, 2:6, 4:4, 6:2, and 7:1, the molar amounts keep at 8 mmol). The synthesis process is similar to pure NiCoP in the manuscript, and the samples are shorted named as NiCoP-1:7, 2:6, 4:4, 6:2, and 7:1. As shown in **Fig. S6**, all obtained samples maintain the nanosheet morphologies. And **Fig. S7** shows the HER and OER performances for NiCoP with different molar ratios of Co/Ni. It can be found that NiCoP sample with the molar ratio of Co:Ni=4:4 shows the best HER and OER performances. Thus, in the following research, we choose NiCoP with the molar ratio of Co:Ni=4:4 for further investigations.

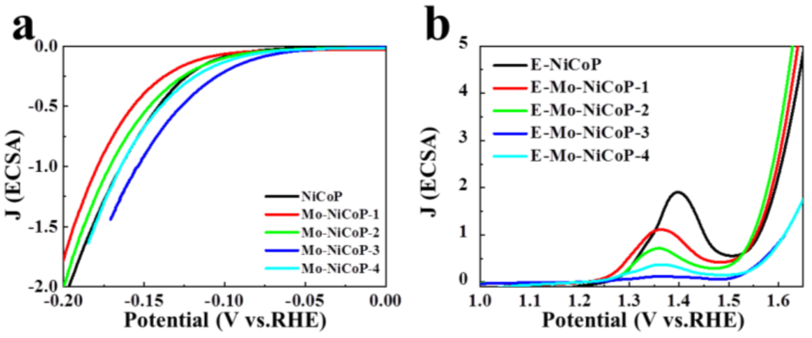

Fig. S8 a HER and **b** OER polarization curves of obtained samples normalized into ECSA

According to latest researches (*ACS Nano 2018, 12, 9635; Nat. Commun. 2018, 9, 2452; Nano Energy 2017, 35, 161.*), the LSV curves of HER and OER are normalized by C_{dl}, using following equations: ECSA = ($C_{dl\text{-}catal.}$ - $C_{dl\text{-}CC}$)/C_s, where a specific capacitance (C_s) value of 0.040 mF cm^{-2} in 1 M KOH was adopted. The normalized LSV curves for HER and OER are shown in **Fig. S8**. For HER, it can be found that Mo-NiCoP-3 sample shows the better performances than that of pure NiCoP, which demonstrates that Mo doping could effectively increase intrinsic activities. It can be found that Mo-NiCoP-1 and Mo-NiCoP-2 show poor performances than that of NiCoP. This phenomenon may be contributed the much increased C_{dl} value or electroactive sites after Mo doping. In other word, the increased electroactive sites may play the main role in the improved performances for Mo-NiCoP-1 and Mo-NiCoP-2.

For OER, it can be found that E-Mo-NiCoP-3 sample do not show the better performances when normalized by C_{dl}. However, E-Mo-NiCoP-2 shows better performances. To some extent, it also demonstrates that Mo doping could effectively increase intrinsic activities for OER. As for E-Mo-NiCoP-3, due to the largest C_{dl} value (89.4 mF cm^{-2}), the improved OER performance is mainly contributed to the richest electroactive sites after Mo doping.

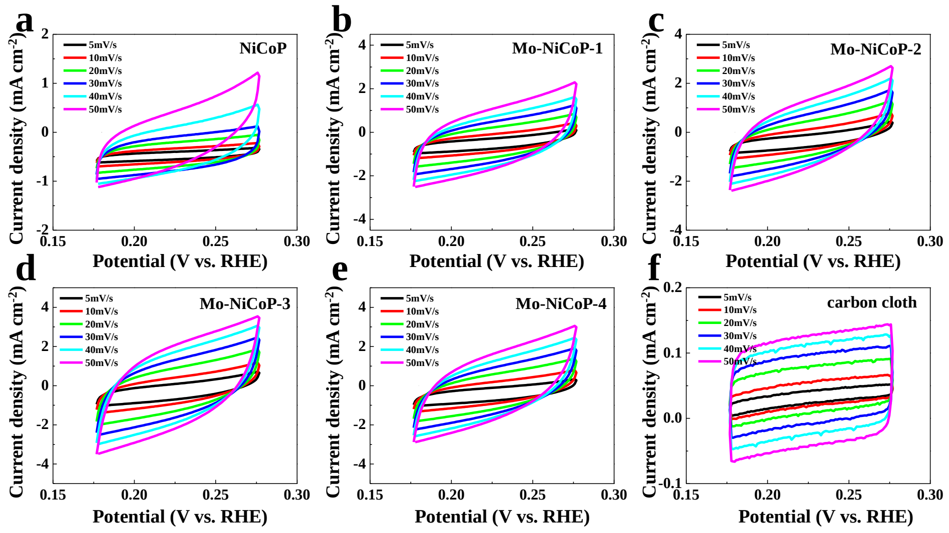

Fig. S9 CV curves of **a** NiCoP, **b** Mo-NiCoP-1, **c** Mo-NiCoP-2, **d** Mo-NiCoP-3, **e** Mo-NiCoP-4, and **f** carbon cloth for estimating the ECSA in HER tests

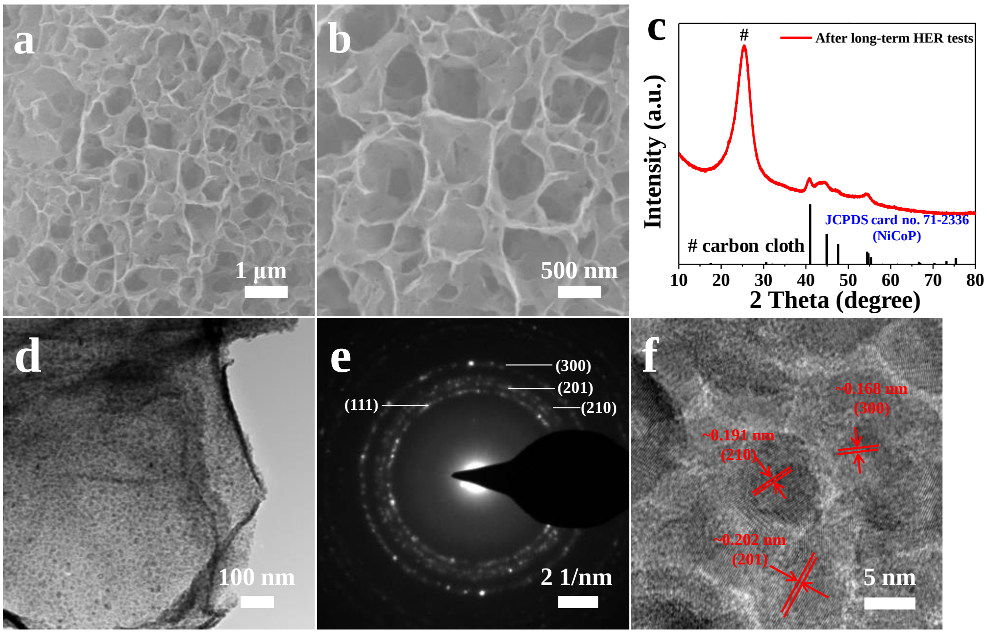

Fig. S10 a, b SEM images, **c** XRD pattern, **d** TEM, **e** SAED and **f** HRTEM images of Mo-NiCoP-3 after long-term HER tests

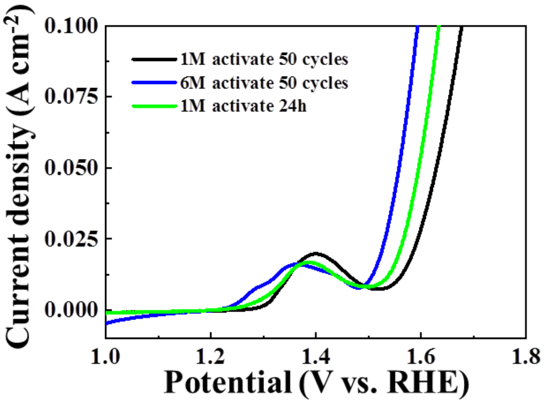

Fig. S11 Polarization curves of E-Mo-NiCoP-3 with different activation cycles as indicated

The initial sample in **Fig. 3a** (in the manuscript) was also activated by 1 M KOH with similar process (50 cycles). To illustrate the key role of 6 M KOH, we also activated sample in 1 M KOH for 24 h. It can be found that the sample activated in 6 M KOH for 50 cycles showed the best OER performances (see **Fig. S11**). Even after 24 h, the OER performance in 1 M KOH is not comparable with that in 6 M KOH. The advantage of choosing 6 M KOH for electrochemical activation could be concluded as following. Firstly, 6 M KOH shows a highest ionic mobility, which is beneficial for electrochemical activation. Secondly, during electrochemical activation, 6 M KOH could fully and fast convert metal phosphides into corresponding metal oxides/hydroxides, which is beneficial for OER reactions. Finally, by an electrochemically induced ion-exchange approach in 6 M KOH, in-situ formed core-branched nanostructures could provide richer electroactive sites, and thus lead to increased OER activity.

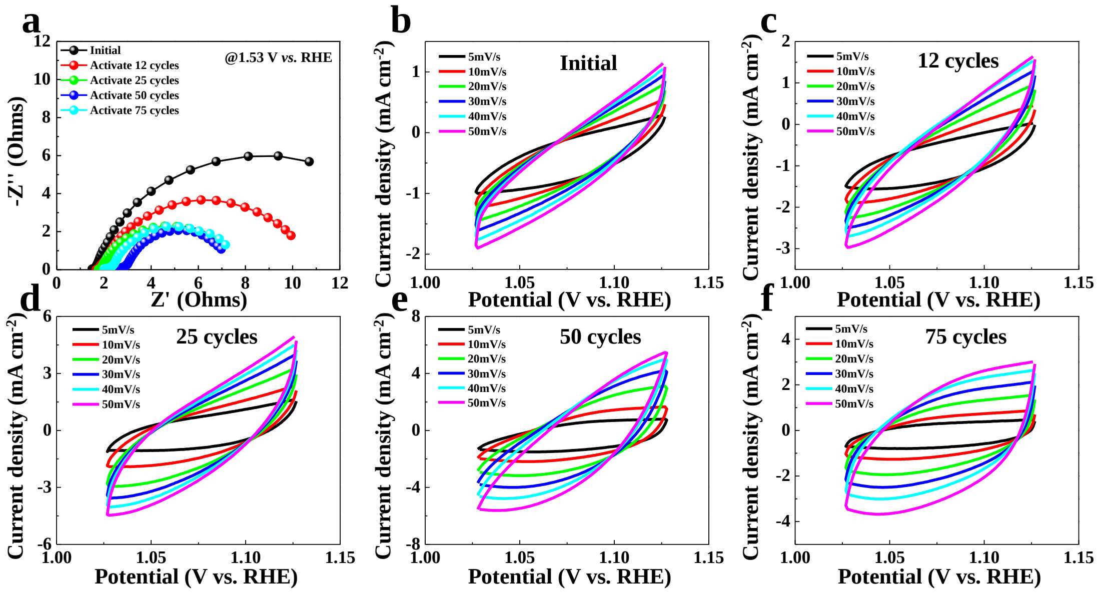

Fig. S12 a The Nyquist plots of E-Mo-NiCoP-3 with different activation cycles. **b-f** CV curves of E-Mo-NiCoP-3 with different activation cycles for estimating the ECSA during the activation process

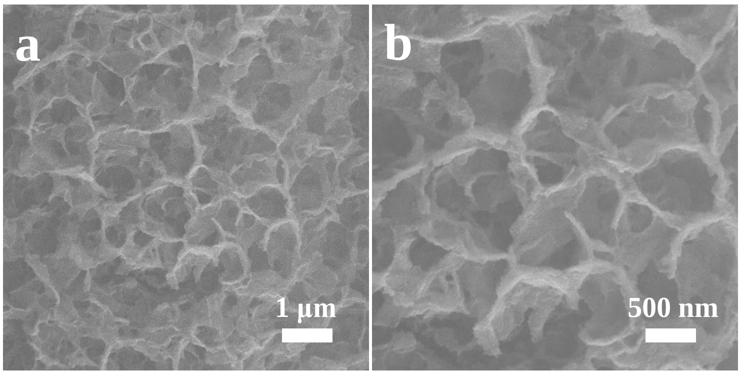

Fig. S13 SEM images of E-NiCoP

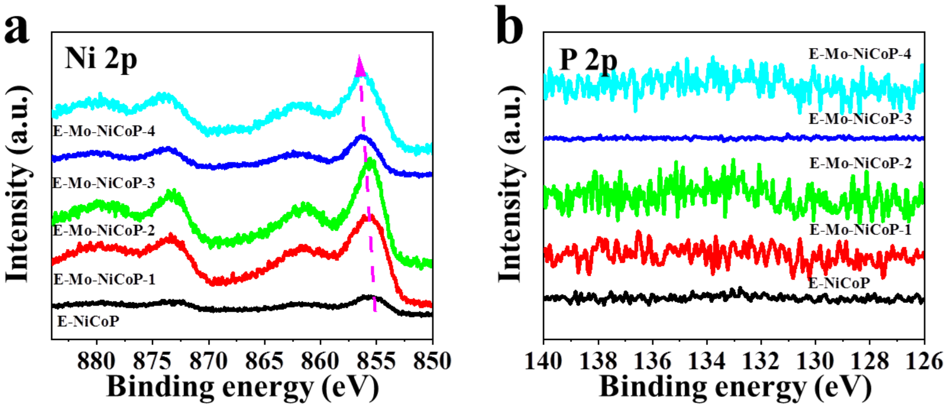

Fig. S14 XPS spectra of **a** Ni 2p and **b** P 2p for all E-Mo-NiCoP

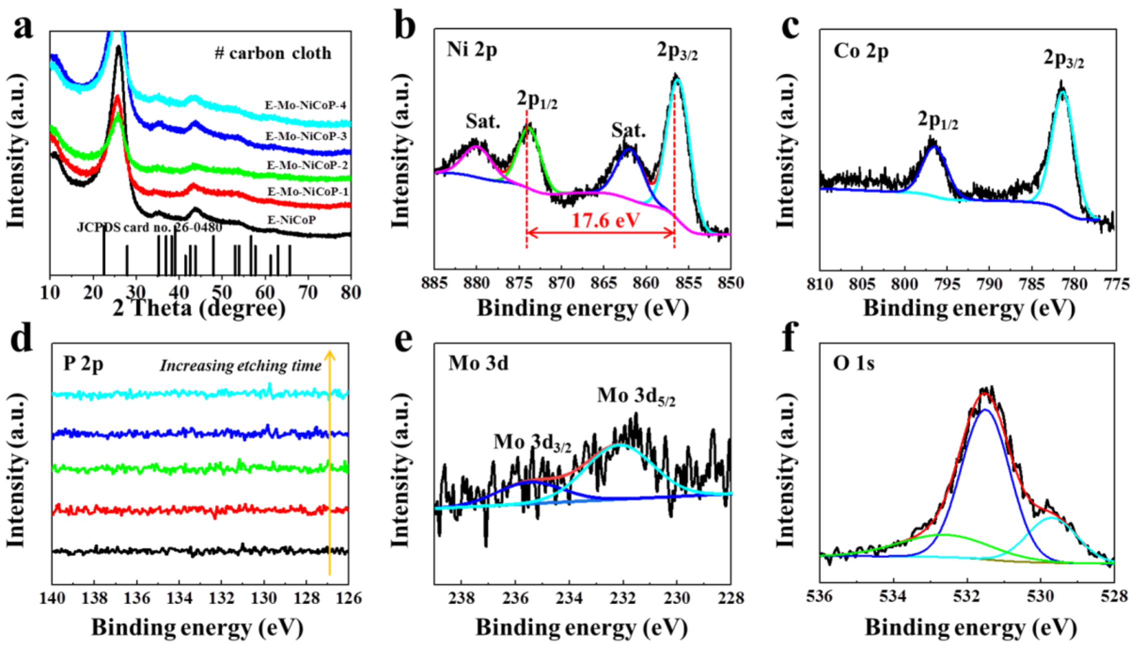

Fig. S15 a XRD patterns of all E-Mo-NiCoP samples. XPS spectra of E-Mo-NiCoP-3: **b** Ni 2p, **c** Co 2p, **d** P 2p, **e** Mo 3d, and **f** O 1s

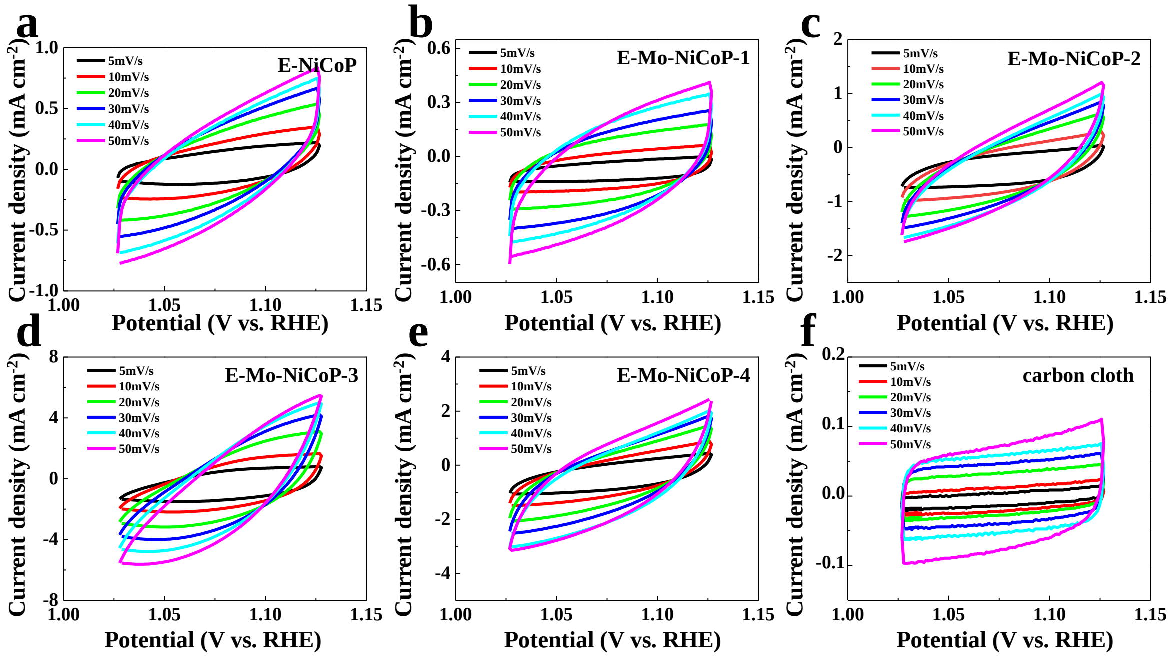

Fig. S16 CV curves of **a** E-NiCoP, **b** E-Mo-NiCoP-1, **c** E-Mo-NiCoP-2, **d** E-Mo-NiCoP-3, **e** E-Mo-NiCoP-4, and **f** carbon cloth for estimating the ECSA in OER tests

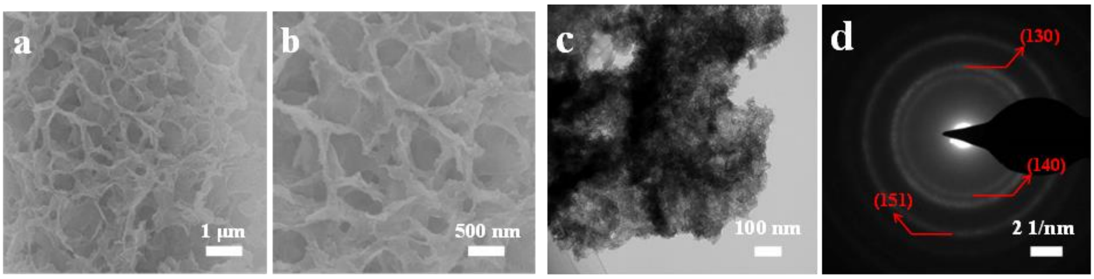

Fig. S17 a, b SEM, **c** TEM, and **d** SAED images of E-Mo-NiCoP-3 after long-term OER tests

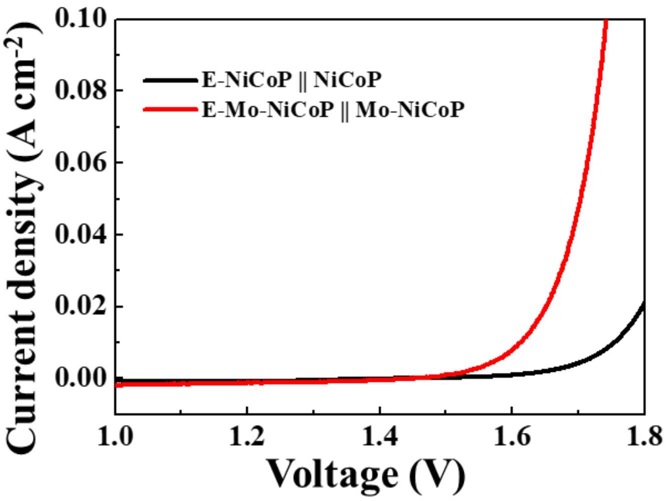

Fig. S18 Overall water splitting of the electrolyzer (E-Mo-NiCoP||Mo-NiCoP) and (E-NiCoP||NiCoP) in 1M KOH solution

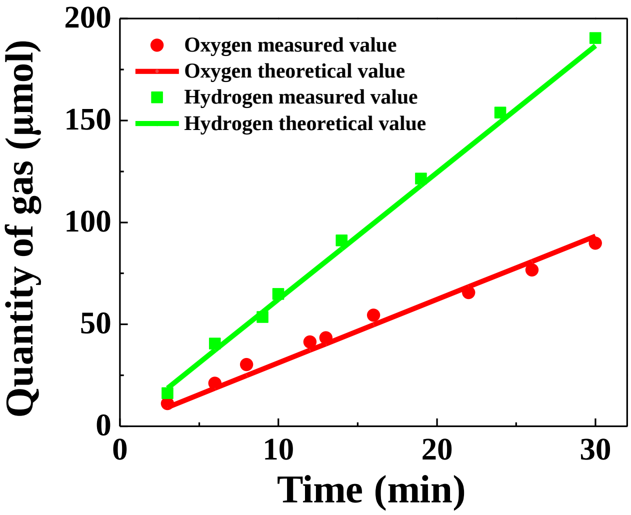

Fig. S19 Amounts of gas calculated and experimentally measured along reaction time for overall water splitting. The theoretical line represents the amount of H_2 or O_2 expected for a 100% Faraday efficiency

Table S1 Comparison of HER performances for Mo-NiCoP nanosheets with reported electrocatalysts in the alkaline media

Electrocatalyst	Substrate	Overpotential (mV)	Tafel slope (mV dec^{-1})	Refs.
Mo-NiCoP	Carbon cloth	76, 121, 148 at 10, 50, 100 mA cm^{-2}	60	**This work**
CoP nanowire by oxygen plasma engraving	Carbon cloth	180 at 100 mA cm^{-2}	42.8	*Adv. Mater.* 2018, **30**, 1703322.
Cobalt Selenide	Co foil	268 at 100 mA cm^{-2}	61.4	*Adv. Energy Mater.* 2018, **8**, 1801926.
Ni_2P/Fe_2P	Ti foil	121, ~210 at 10, 100 mA cm^{-2}	67	*Adv. Energy Mater.* 2018, **8**, 1800484
Ni_2P-Ni_3S_2	Ni foam	80, ~175 at 10, 100 mA cm^{-2}	65	*Nano Energy* 2018, **51**, 26.
WS_2/Ni_5P_4-Ni_2P	Ni foam	94, 211 at 10, 100 mA cm^{-2}	74	*Nano Energy* 2019, **55**, 193.
$Co_5Mo_{1.0}P$ nanoshets	Ni foam	173, 300 at 10, 100 mA cm^{-2}	190.1	*Nano Energy* 2018, **45**, 448.
Hyperbranched NiCoP Arrays	Ni foam	71, 155 at 10, 100 mA cm^{-2}	57	*ACS Appl. Mater. Interfaces* 2018, **10**, 41237.
NiCoP nanocone	Ni foam	104, 197 at 10, 100 mA cm^{-2}	54	*J. Mater. Chem. A* 2017, **5**, 14828.
Ni-Co-P hollow nanobricks	Powder	107, ~185 at 10, 100 mA cm^{-2}	46	*Energy Environ. Sci.* 2018, **11**, 872.
Mo-doped Ni_3S_2 nano-rods	Ni foam	180 at 100 mA cm^{-2}	72.9	*J. Mater. Chem. A* 2017, **5**, 1595.
N-Ni_3S_2	Ni foam	110, ~240 at 10, 100 mA cm^{-2}	-	*Adv. Mater.* 2017, **29**, 1701584.
$Co_{0.93}Ni_{0.07}P_3$ nanoneedle array	Carbon cloth	87 at 10 mA cm^{-2}	60.7	*ACS Energy Lett.* 2018, **3**, 1744.
NC/CuCo/CuCoOx nanowires arrays	Ni foam	112, 190 at 10, 100 mA cm^{-2}	55	*Adv. Funct. Mater.* 2018, **28**, 1704447
$TiO_2@Co_9S_8$ core-branch arrays	Ni foam	139, ~190 at 10, 50 mA cm^{-2}	65	*Adv. Sci.* 2018, **5**, 1700772
NiFe LDH@NiCoP	Ni foam	120, ~230, ~320 at 10, 50, 100 mA cm^{-2}	88.2	*Adv. Funct. Mater.* 2018, **28**, 1706847.
Fe doped Ni_3S_2	Ni foam	47, 232 at 10, 100 mA cm^{-2}	95	*ACS Catal.* 2018, **8**, 5431.

Notes: 1. If not metioned specifically, all overpotentials were corrected with iR compensation. 2. If not metioned specifically, all electrocatalysts are directly synthesized on conductive substrates.

Table S2 Comparison of OER performances for E-Mo-NiCoP nanosheets with reported electrocatalysts in the alkaline media

Electrocatalyst	Substrate	Overpotential (mV)	Tafel slope (mV dec^{-1})	Refs.
E-Mo-NiCoP	Carbon cloth	269, 328, 364 at 10, 50, 100 mA cm^{-2}	76.7	**This work**
$NiCo_2P_2$/graphene quantum dot	Ti mesh	400 at 100 mA cm^{-2}	65.9	*Nano Energy* 2018, **48**, 284.
plasma-assisted synthesized NiCoP	Ni foam	280 at 10 mA cm^{-2}	87	*Nano Lett.* 2016, **16**, 7718.
CoS_2 nanotube	Carbon cloth	276 at 10 mA cm^{-2}	81	*Nanoscale Horiz.* 2017, **2**, 342.
Hyperbranched NiCoP Arrays	Ni foam	268, 350 at 10, 100 mA cm^{-2}	75	*ACS Appl. Mater. Interfaces* 2018, **10**, 41237.
NiO@Ni/WS_2	Carbon cloth	380 at 50 mA cm^{-2}	108.9	*ACS Cent. Sci.* 2018, **4**, 112.
N-$NiMoO_4$/NiS_2	Carbon cloth	267, 335 at 10, 100 mA cm^{-2}	44.3	*Adv. Funct. Mater.* 2019, **29**, 1805298.
Mo-CoOOH	Carbon cloth	305, 365 at 10, 100 mA cm^{-2}	56	*Nano Energy* 2018, **48**, 73.
$Co_5Mo_{1.0}O$ nanosheets	Ni foam	270, 330 at 10, 100 mA cm^{-2}	54.4	*Nano Energy* 2018, **45**, 448.
Mo-NiOOH	Ni foam	390 at 100 mA cm^{-2}	68	*Int. J. Hydrogen Energy* 2018, **43**, 12140.
NiCoP cone shaped nanowire	Ni foam	370 at 100 mA cm^{-2}	116	*J. Mater. Chem. A* 2017, **5**, 14828.
$NiMoP_2$ nanowires	Carbon cloth	330 at 100 mA cm^{-2}	90.6	*J. Mater. Chem. A* 2017, **5**, 7191.
Ni_3S_2@MoS_2/FeOOH	Ni foam	260 at 10 mA cm^{-2}	49	*Appl. Catal. B* 2019, **244**, 1004.
C@Ni_8P_3	Ni foam	267 at 10 mA cm^{-2}	51	*ACS Appl. Mater. Interfaces* 2016, **8**, 27850.

Notes: 1. If not metioned specifically, all overpotentials were corrected with iR compensation. 2. If not metioned specifically, all electrocatalysts are directly synthesized on conductive substrates.

Table S3 Comparison of water-splitting performances for E-Mo-NiCoP||Mo-NiCoP with reported bifunctional electrocatalysts in the alkaline media

Electrocatalyst	Substrate	Potential	Refs.
E-Mo-NiCoP||Mo-NiCoP	Carbon cloth	1.61 V at 10 mA cm^{-2}	**This work**
$NiCo_2P_2$/graphene quantum dot	Ti mesh	1.61 V at 10 mA cm^{-2}	*Nano Energy* 2018, **48**, 284.
P-Co_3O_4	Ni foam	1.63 V at 10 mA cm^{-2}	*ACS Catal.* 2018, **8**, 2236.
$NiMoP_2$ nanowires	Carbon cloth	1.67 V at 10 mA cm^{-2}	*J. Mater. Chem. A* 2017, **5**, 7191.
CoS_2 nanotube	Carbon cloth	1.67 V at 10 mA cm^{-2}	*Nanoscale Horiz.* 2017, **2**, 342.
N-$NiMoO_4$/NiS_2	Carbon cloth	1.60 V at 10 mA cm^{-2}	*Adv. Funct. Mater.* 2019, **29**, 1805298.
FeCoP ultrathin arrays	Ni foam	1.60 V at 10 mA cm^{-2}	*Nano Energy* 2017, **41**, 583.
$Co_5Mo_{1.0}O$//$Co_5Mo_{1.0}P$ nanosheets	Ni foam	1.68 V at 10 mA cm^{-2}	*Nano Energy* 2018, **45**, 448.
Ni/Mo_2C	Powder	1.66 V at 10 mA cm^{-2}	*Chem. Sci.* 2017, **8**, 968.
